# Assessment of novel POCT to evaluate liver function

**DOI:** 10.1016/j.plabm.2024.e00367

**Published:** 2024-01-30

**Authors:** Carmen Minea, Damien Gruson

**Affiliations:** a Department of Clinical Biochemistry, Hospital Universitario Fundación Jiménez Díaz, Madrid, Spain; b Department of Clinical Biochemistry, Cliniques Universitaires St-Luc, Université Catholique de Louvain, Brussels, Belgium; c Pôle de recherche en Endocrinologie, Diabète et Nutrition, Institut de Recherche Expérimentale et Clinique, Cliniques Universitaires St-Luc, Université Catholique de Louvain, Brussels, Belgium

**Keywords:** POCT, Liver function

## Abstract

**Objectives:**

Point of care testing (POCT) offers the possibility of near bedside patient testing with a reduction of the turn-around time of analysis. The aim of our study was to determine the analytical performances and usability of a recently developed POCT device for the measurement of tests related to liver function. We evaluated the performance of a liver tests panel performed on the LINX EVO® POCT device.

**Design and methods:**

The imprecision was determined with the Bio-Rad Liquichek Unassayed Chemistry Control. Method comparison was performed with a Cobas® 8000 analyzer. Samples from twenty healthy volunteers were used to verify the reference intervals. Furthermore, practicality was assessed by the healthcare staff handling the POCT device through a dedicated questionnaire.

**Results:**

The imprecision observed was matching the criteria for the in-lab assay with only one exception, globulin, with an observed imprecision of 6.3 % and a criteria of 5.7 %. With the exception of total and direct bilirubin, the POCT method showed good agreement with the in-lab methods. The verification of reference intervals showed that more than 90 % of the healthy volunteer values were included into the reference interval claimed by the manufacturer except for glucose and globulin. The POCT practicality questionnaire was satisfying overall for users.

**Conclusions:**

Our study showed very good analytical performances overall for the liver test panel performed on the LINX EVO® POCT instrument.

## Highlights

• Novel panel of point of care tests to evaluate liver function in 15 min.
• The imprecision observed was matching the criteria with the exception of globulin.
• Method comparison showed an excellent agreement with the routine laboratory method for the majority of the parameters.

## 1 Introduction

Nowadays Point-of-Care Testing (POCT) devices are living proof that science and technology are growing and blending together to offer reliable results outside of a laboratory environment. The analytical method is simplified for relatively quick results, with the big advantage that personnel without formal laboratory training can manage the devices, even outside of the hospital settings [1,2]. Therefore, apart from a precise analytical performance, user-friendliness and ease of use are also important characteristics of a POCT device [3,4]. Increasingly, more POCT devices are designed to not only offer one parameter but panels with different parameters that permit complex medical decisions and specific monitorization and in this specific situation the POCT must offer reliable results [5]. The aim of this study was to evaluate the analytical performance of a recently developed POCT device to evaluate liver function.

## 2 Material and methods

The novel LINX EVO® POCT Instrument (Menarini Diagnostics) is a POCT device that offers the possibility of testing several parameters simultaneously for measurement of markers of liver function within 15 min, using colorimetric methods. The panel includes different parameters such as: Albumin, Alkaline Phosphatase, Aspartate Aminotransferase (AST), Alanine Aminotransferase (ALT), Gamma Glutamyl Transpeptidase, Direct Bilirubin, Total Bilirubin, Glucose, Total Protein, Globulin, Indirect Bilirubin. Indirect Bilirubin is a calculated parameter between the total and direct bilirubin. For a comprehensive and panoramic assessment of this POCT instrument we evaluated various aspects: imprecision characteristics, verification of the reference intervals, method comparison with the routine laboratory method (Cobas® 8000 analyzer) and user-friendliness.

### 2.1 Analytical performances

The imprecision was assessed by running Bio-Rad Liquichek Unassayed Chemistry Control, twice daily, over 5 days [6]. The coefficient of variation (CV) was determined based on the between-day variation.

The verification of the reference intervals was conducted using serum and plasma samples after prior identification of 20 healthy volunteers [7].

For the method comparison with Cobas® 8000 analyzer, 25 samples of heparin plasma were used [7,8]. All the samples were left-over from the emergency room and anonymized. The research related to human use complied with all the relevant national regulations, institutional policies and in accordance with the tenants of the Helsinki Declaration.

### 2.2 User-friendliness

A secondary outcome of our study was the evaluation of the user-friendliness of the POCT analyzer. 6 clinical trainees (laboratory technicians in training, laboratory medicine doctors in training, pharmacy trainees, last year medicine students with a special interest in Clinical Biochemistry), all first-time users, completed the French version of the questionnaire [9]. The questionnaire had various items that refer to different aspects of the POCT characteristics: a) rating of the information in the manual, b) rating of operation facilities, c) rating of time factors, d) rating of quality control procedure. The answers permit that the ten items were classified as satisfactory (S), intermediary (I) and unsatisfactory (U). The final part of the questionnaire offers the possibility for users to write freely their own impressions about the device.

### 2.3 Statistical analysis and interpretation

The correlation was analyzed and plotted using the Passing–Bablok Regression Method and the differences and agreement according to the Bland–Altman Method. Computations were performed using XLSTAT 2020.

## 3 Results

The CV was calculated for each parameter included in the liver panel (Table 1). These results agreed with the Total Error (TE) specified in the Westgard database [10]. The only exception was globulin with an observed imprecision of 6.3 % and a criteria of 5.7 %. When performing the verification of reference intervals, only glucose and globulin displayed values outside of the reference intervals claimed by the manufacture.

**Table 1:** Imprecision of the liver tests panel of the LINX EVO® POCT instrument.

	Albumin	ALP	AST	ALT	Direct bilirubin	Total Bilirubin	GGT	Glucose	Total Protein	Globulin	Indirect bilirubin
CV% Imprecision^a^	1.55	2.56	4	4.63	0	6.8	4.28	1.3	0.93	6.3	18.57
Imprecision% Westgard Table (I)^b^	1.6	3.23	6.15	9.7	18.4	10.90	6.7	2.8	1.38	5.7	–
Total Error % Westgard Table (TE)^b^	4.07	12.4	16.69	27.48	44.5	26.94	22.11	6.96	3.63	8	–

a The Imprecision was assessed using Bio-Rad Liquichek Unassayed Chemistry Control, twice daily, during 5 days was.

b As specify for I (%) and TE (%) on https://www.westgard.com/biodatabase1.htm.

The Bland-Altman agreement method and Passing and Bablok regression are presented in Table 2. When reviewing Passing and Bablok and Bland and Altman plots, we noticed several differences between the two techniques in the low range of values in the case of total and direct bilirubin, but despite the differences, the results were still within normal range. Although this difference was seen with one or two results in the rest of the parameters, none of the results made a difference in the clinical interpretation, meaning that none of the results were classified as normal when it was in the pathological range or the other way around. A high result was still a high pathological result for both techniques. For the comparison method, globulin was not included as this technique is not available on the Cobas® 8000. Nevertheless, the total value of globulins is not a common parameter used in clinical practice as specific types of globulins are more frequently used.

**Table 2:** Bland-Altman agreement method and Passing- Bablok regression.

	Correlation Coefficient (R squared)	Bias 95 %IC	Slop 95 % IC	Intercept 95 %CI	Number of tests
Albumin	0.972	2.164 (−0.70 to 5.02)	0.833 (0.763–0.909)	9.083 (6.00–12.153)	25
ALP	0.986	−2.764 (25.20–19.6)	0.783 (0.722–0.834)	15.350 (12.124–22.732)	25
ALT	0.986	12.98 (−3.94 to 29.90)	1.22 (1.037–1.342)	7.111 (4.466–11.185)	25
AST	0.959	4.229 (−2.77 to 11.23)	0.879 (0.667–0.981)	7.098 (4.642–12)	25
Direct bilirubin	0.578	−0.001 (−0.15 to 0.15)	0.896 (0.526–1.154)	0.031 (−0.019 to 0.105)	25
Total Bilirubin	0.181	−0.01 (−0.51 to 0.49)	0 (0.00–0.156)	0.400 (0.367–0.400)	25
GGT	0.999	9.818 (−33.28 to 52.92)	1.199 (1.130–1.249)	−1.196 (−3.107 to 1.236)	25
Glucose	0.882	−7.804 (−32.30 to 16.70)	0.950 (0.734–1.128)	2.472 (−16.780 to 18.510)	25
Total Protein	0.967	1.9 (−25.35 to 29.15)	1.026 (0.922–1.114)	2.703 (−8.555 to 4.499)	25

The user-friendliness of the POCT was overall satisfying and most of the answers tilt the scale towards Satisfactorÿ especially regarding those items related to the information in the manual, operation facilities and time factors. The comments added by users in the last part of the questionnaire were positive overall, such as the device being intuitive and easy to use, available interface languages, the touchscreen and the rapidity of measurements.

## 4 Discussion

A POCT device that offers different types of liver function parameters so quickly may be highly valuable in a hospital setting, clinics and primary care where POCT are being incorporated and can open a tool for personalized medicine [[11], [12]]. Our study is the first to explore the analytical reliability and usability of the POCT liver tests panel.

Our results showed a good overall analytical performance for the liver panel of the LINX EVO® POCT instrument. The imprecisions that we observed were matching the targets found in Westgard database. Furthermore, the method comparison showed a good agreement with the Cobas® 8000 central lab analyzer. Even if a few differences between both methods was observed, this didn't change clinical decisions, as they were not found to be clinically significant. Some of the differences can be attributed to a difference in the limits of detection of the two methods. For example, if the total bilirubin result was lower than 0.4 mg/dL with the central lab analyzer, the POCT instrument would read <0.4 mg/dL. The difference in sensitivity was also seen for direct bilirubin, and it was even more visible with AST where the POCT system displayed results as <20 U/L and the central lab analyzer informed results as low as 11 U/L.

Our study also aimed to verify the reference intervals of the POCT liver panel [5]. The reference intervals provided by the manufacturer were confirmed with the exceptions of glucose and globulin. Manufacturer reference interval for glucose is a fasting reference interval, were in healthy volunteers fasting prior the testing was not assessed. For globulin, the results were very close to the inferior limit of the reference interval provided by the manufacturer. Nevertheless, globulin is not a clinically relevant parameter in assessing the liver function. Gender specific reference intervals were suggested by the manufacturer only for the GGT.

The results of our evaluation also confirmed the usability of the POCT system, which is also very important insofar as the healthcare staff who can use these instruments are not always familiar with laboratory devices and methods [13].

To overcome the limited bias with routine laboratory assays, it is recommended that institutions verify the reference intervals in their local patient population.

In conclusion, our study showed the overall good analytical performance for the liver tests panel performed on the LINX EVO® POCT instrument. The usability of the system was also confirmed.

## Conflict of interest disclosure statement

This research did not receive any specific grant from funding agencies in the public, commercial, or not-for-profit sectors.

## CRediT authorship contribution statement

**Carmen Minea:** Data curation, Formal analysis, Writing – original draft. **Damien Gruson:** Conceptualization, Supervision, Writing – review & editing.

## Declaration of competing interest

We confirm that this work is original and has not been published elsewhere, nor is it currently under consideration for publication elsewhere.

In this paper, we report on a new POCT device that offers quick results of several parameters of the liver function. This is significant because aassuring the quality of the results of POCT devices should be provided by the laboratory personnel already skilled in the field.

We believe that this manuscript is appropriate for publication by PRACTICAL LABORATORY MEDICINE because it focuses on analytical performance oof a point of care tests panel that can be used for diagnosis, prognosis, treatment and therapy, and monitoring of disease.

We have no conflicts of interest to disclose. We have not receive relevant financial support (for example patent ownership, stock ownership, consultancies, and speaker's fees). And we don't have any personal, political, intellectual, or religious interests.

## Data Availability

Data will be made available on request.
