# Supplementary material for: Assessment of novel POCT to evaluate liver function
Source: Pract Lab Med. 2024 Jan 30;39:e00367. doi: 10.1016/j.plabm.2024.e00367 (PMC10847987; doi:10.1016/j.plabm.2024.e00367)
Supplement: Multimedia component 1 [file mmc1.docx]

Supplemental data of the manuscript

Assessment of novel POCT to evaluate liver function.

Carmen Minea¹, Damien Gruson²'³

*^1^Department of Clinical Biochemistry, Hospital Universitario Fundación Jiménez Díaz, Madrid, Spain.*

^2^*Department of Clinical Biochemistry, Cliniques Universitaires St-Luc, Université Catholique de Louvain, Brussels, Belgium.*

^3^*Pôle de recherche en Endocrinologie, Diabète et Nutrition, Institut de Recherche Expérimentale et Clinique, Cliniques Universitaires St-Luc, Université Catholique de Louvain, Brussels, Belgium.*

Correspondence:

Pr. Damien Gruson,

*Department of Laboratory Medicine*

*Cliniques Universitaires St-Luc and Université Catholique de Louvain*

10 Avenue Hippocrate,

B-1200 Brussels, Belgium

Phone +32-(0)2-7646747, fax +32-(0)2-7646930

Email: [damien.gruson@uclouvain.be](mailto:damien.gruson@uclouvain.be)

**Table S1.** Reference intervals of the liver tests panel of the LINX EVO^®^ POCT instrument

|  | **Albumin** | **ALP** | **AST** | **ALT** | **Direct**  **bilirubin** | **Total**  **Bilirubin** | **GGT** | **Glucose** | **Total**  **Protein** | **Globulin** | **Indirect**  **bilirubin** |
| --- | --- | --- | --- | --- | --- | --- | --- | --- | --- | --- | --- |
| **Percentage of values ​​out of range** | < 10% | < 10% | <10% | <10% | < 10% | < 10% | < 10% | >10% | < 10% | >10% | < 10% |
| **Numbers of values out of range** | (1)  55 g/L | (0) | (0) | (0) | (1)  0.6mg/L | (0) | (1)  82U/L | (3)  56 mg/dL  67 mg/dL  134mg/dL | (1)  84 g/L | (4)  20 g/L  20 g/L  21 g/L  21 g/L | (0) |
| **Reference Intervals *** | 35-53 g/L | <108  U/L | <40  U/L | <40  U/L | <0.4 mg/dL | <1.2  mg/dL | M: <73U/L  F: <38 U/L | 70-110 mg/dL | 60-83 g/L | 23-35  g/L | 0.2-0.8mg/dL |

*Reference intervals claimed by the manufacturer

**Table S2.** User-friendliness of the POCT questionnaire

| **Criteria** | **Satisfactory**  **(S)** | **Intermediary (I)** | **Unsatisfactory**  **(U)** | **Results** |
| --- | --- | --- | --- | --- |
|  |  |  |  |  |
| **Device weight** | <5kg | 5kg < x < 10kg | 10 kg < | SSSS  II |
| **Device size** | <5cm x 5cm | 5cm x 5cm < x < 10cm x 10cm | 30cm x 30cm < | IIIIII |
| **Additional equipment needed to perform the analysis (device, cartridges, consumables)** | No | 1-2 | >3 | UUUUUU |
| **Description of the sample collection process** | Yes | Incomplete | No | SSSSSS |
| **Description of the test process** | Yes | Incomplete | No | SSSSSS |
| **Description of how the results are presented (and the units used are the same as those used for the routine testing)** | Yes | Incomplete | No | SSSSSI |
| **Function and use of the test (in witch cases it can be used: diagnosis / risk prediction / monitoring)** | Yes | Incomplete | No | SSSSII |
| **Presence of the necessary instructions for the user to perform the test correctly** | Yes | Incomplete | No | SSSSSS |
| **Training required to collect samples** | No | Incomplete | Yes | SSSSSS |
| **Training required to use the device** | No | Basic training | Special training | SSSSII |
| **Training necessary for the calibration and quality control of the device (presence of instructions to carry them out)** | Yes | Incomplete | No | SSIUU |
